# Role of Lysophospholipid Metabolism in Chronic Myelogenous Leukemia Stem Cells

**DOI:** 10.3390/cancers13143434

**Published:** 2021-07-08

**Authors:** Kazuhito Naka

**Affiliations:** Department of Stem Cell Biology, Research Institute for Radiation Biology and Medicine, Hiroshima University, 1-2-3 Kasumi, Minami-ku, Hiroshima 734-8553, Japan; kanaka55@hiroshima-u.ac.jp; Tel./Fax: +81-82-257-5808

**Keywords:** CML stemness, lysophospholipid, Gdpd3, Foxo3a

## Abstract

**Simple Summary:**

In this review, I discuss our recent finding that lysophospholipid metabolism is essential for the maintenance of chronic myelogenous leukemia (CML) stem cells. Lysophospholipids have only one fatty acid chain and so are more hydrophilic than phospholipids, allowing them to act as lipid second messengers. We demonstrated that the stem cell quiescence and TKI resistance displayed by CML stem cells in vivo are sustained by the Gdpd3 enzyme involved in lysophospholipid metabolism. At the mechanistic level, Gdpd3 function allows lysophospholipid metabolism to suppress the AKT/mTORC1-mediated cell growth pathway while activating the stemness factors FOXO and β-catenin. Our results thus link lysophospholipid metabolism to CML stemness, and may thereby open up new therapeutic avenues to overcome CML relapse post-TKI therapy.

**Abstract:**

It is well known that mature chronic myelogenous leukemia (CML) cells proliferate in response to oncogenic BCR–ABL1-dependent signaling, but how CML stem cells are able to survive in an oncogene-independent manner and cause disease relapse has long been elusive. Here, I put into the context of the broader literature our recent finding that lysophospholipid metabolism is essential for the maintenance of CML stem cells. I describe the fundamentals of lysophospholipid metabolism and discuss how one of its key enzymes, Glycerophosphodiester Phosphodiesterase Domain Containing 3 (Gdpd3), is responsible for maintaining the unique characteristics of CML stem cells. I also explore how this knowledge may be exploited to devise novel therapies for CML patients.

## 1. Introduction

### 1.1. CML Stem Cells in CML Disease

Chronic myeloid leukemia (CML) arises when the *BCR–ABL1* fusion oncogene forms and is activated in hematopoietic stem cells (HSCs) [1,2]. CML stem cells bearing this oncogene are generated and in turn give rise to most mature CML cells, which also bear this aberration. The pro-proliferative signaling triggered by *BCR–ABL1* can be blocked by tyrosine kinase inhibitors (TKIs), which has dramatically improved the prognosis of many CML patients [2]. The first such TKI was imatinib mesylate (imatinib), and second-generation TKIs include dasatinib, nilotinib and bosutinib. Indeed, about 40–70% of chronic phase (CP)-CML patients who show a deep molecular response (DMR) to TKI therapy enjoy significant relapse-free survival without further TKI treatment [3,4,5]. However, the remaining 30–60% of CML patients who exhibit a DMR unfortunately suffer a relapse of CML disease after discontinuing TKI treatment. A major question in the field has thus been “What distinguishes CML patients who relapse from those who don’t?”.

It turns out that the CML stem cells that generate most mature CML cells are responsible for disease relapse post-TKI therapy [6,7,8]. While mature CML cells actively proliferate due to BCR–ABL1-dependent signaling, CML stem cells are able to avoid proliferation and maintain quiescence in an oncogene-independent fashion [9]. TKIs therefore eliminate most proliferating mature CML cells but have no effect on the quiescent CML stem cells [10,11]. After TKI therapy is stopped, the surviving CML stem cells emerge from quiescence and give rise to a new cadre of mature CML cells. To date, much research effort has been devoted to investigating the molecular mechanisms by which CML stem cells exploit stem cell quiescence, and searching for new modes of therapy that can be combined with TKI therapy to eradicate not only mature CML cells but also CML stem cells. Many researchers reported that numerous molecular mechanisms regulate the quiescence and TKI resistance in CML stem cells in vivo [6,7,8]. It is reportedly known that several factors within the bone marrow microenvironmental niche are also responsible for the maintenance of self-renewal capacity in CML stem cells in a non-cell autonomous manner [6,7,8].

### 1.2. Transcriptional Control in CML Stem Cells

To date, it is reportedly known that several signaling pathways maintain the self-renewal capacity in CML stem cells, such as JAK/STAT, Hedgehog, Wnt/β-catenin, PI3K–Akt and TGF-β-FOXO signaling within the bone marrow microenvironmental niche [6,7,8]. Pellicano et al. and our group reported that the forkhead O transcription factor FOXO plays an essential role in the maintenance of human and murine CML stem cells [12,13]. In mature CML cells, proliferation is promoted by activation of the PI3K–Akt signaling pathway that is triggered by BCR–ABL1. Activated Akt phosphorylates nuclear Foxo3a, which is then exported to the cytoplasm. This export suppresses Foxo3a’s transcriptional capacity in mature CML cells. In contrast, in quiescent CML stem cells, Akt is inactive despite the presence of BCR–ABL1 and Foxo3a remains within the nucleus and drives transcriptional activity [12,13]. The question then arises: What is the factor that overcomes BCR–ABL1 and suppresses Akt activation in CML stem cells such that Foxo3a is allowed to function? Our investigation revealed that lysophospholipid metabolism inhibits Akt, and that it is the lysophospholipase D enzyme Glycerophosphodiester Phosphodiesterase Domain Containing 3 (Gdpd3) that plays an essential role in maintaining stem cell quiescence and TKI resistance in CML stem cells [14,15]. This involvement of lysophospholipid metabolism in CML stemness opens up a new field of investigation in the realm of novel CML treatments. In this review, I attempt to familiarize the reader with the biological fundamentals of lysophospholipid metabolism, and to highlight ways in which these mechanisms might be targeted as fresh avenues of therapy for CML patients.

### 1.3. Biology of Lysophospholipids and Lysophosphatidic Acids

**Figure 1:**
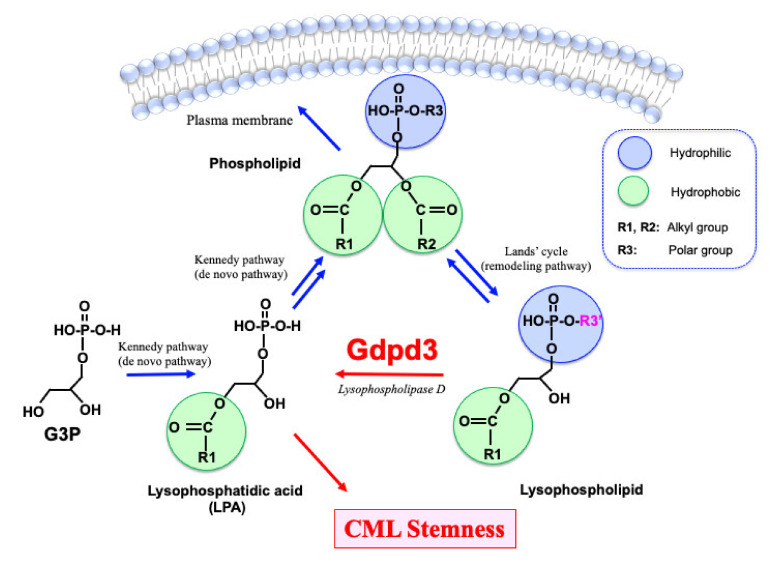
Lipid metabolism in CML stem cells. Glycerophospholipids (phospholipids), which organize the lipid bilayer in a cell’s plasma membrane, are synthesized from lysophosphatidic acids (LPAs). LPAs originate from glycerol 3-phosphate (G3P) via the Kennedy pathway (de novo pathway). Phospholipids are converted to lysophospholipids via the Lands’ cycle (remodeling pathway), which can reverse to produce a wide variety of phospholipids. Lysophospholipids are recycled back into LPAs by lysophospholipase D enzymes such as Gdpd3. Whereas phospholipids have two hydrophobic fatty acid chains, lysophospholipids and LPAs have only one fatty acid chain. Thus, lysophospholipids and LPAs are more hydrophilic than phospholipids and can act as lipid second messengers. We recently demonstrated that CML stemness in vivo depends on Gdpd3 and its function in lysophospholipid metabolism [14].

The lipid bilayer in the plasma membrane of most cells consists of the familiar glycerophospholipids (commonly referred to as “phospholipids”) that contain two fatty acid ester chains and one polar group (Figure 1). In contrast, lysophospholipids and lysophosphatidic acids (LPAs) have only one fatty acid ester chain and are more hydrophilic than phospholipids. Indeed, LPAs are not only intermediates in phospholipid biosynthesis but can also act as “lipid second messengers” themselves. With respect to phospholipid biosynthesis, glycerol 3-phosphate (G3P) is first converted into LPAs and then into various phospholipids via the Kennedy pathway (the so-called de novo pathway) [16] (Figure 1). The fatty acid ester chains and polar bases of phospholipids can then be chemically substituted via the Lands’ cycle (remodeling pathway) to sustain the production of a wide variety of phospholipid molecules [17,18]. Lysophospholipids are recycled back into LPAs by lysophospholipase D enzymes. To date, three lysophospholipase D enzymes, namely Autotaxin (ATX), GDPD3 (also termed GDE7) and GDPD1 (GDE4), have been shown to specifically hydrolyze the polar base of lysophospholipids, such as choline, ethanolamine, inositol and serine [19,20,21]. Recently, we reported that Gdpd3 plays an essential role in maintaining CML stemness in vivo [14].

## 2. Biological Significance of Gdpd3 in CML Stem Cells

### 2.1. Stem Cell Quiescence and TKI Resistance

To investigate gene expression changes specific to CML stem cells, we performed a comparative RNA-Seq analysis of murine normal HSCs and CML stem cells. We found that the most primitive long-term (LT) CML stem cells expressed the *Gdpd3* gene more highly than normal LT-HSCs [14]. While lysophospholipase D enzymes were recycled back into LPA from lysophospholipids, a biological role of Gdpd3 was not identified. We thus established a *Gdpd3*-deficient mouse strain using genome-editing methodologies. Then, we established a CML-like disease model by retroviral *BCR–ABL1* transduction into HSCs isolated from WT and *Gdpd3*-deficent mice, followed by bone marrow transplantation (BMT) into recipient mice. We isolated CML stem cells from these CML-affected mice and evaluated their self-renewal capacity in serial BMT experiments in vivo. After a first-round of BMT, mice that were transplanted with CML stem cells from *Gdpd3*-deficient mice (*Gdpd3*-deficient CML stem cells) and developed CML disease succumbed more rapidly than recipients transplanted with CML stem cells from WT mice (WT-CML stem cells). We then purified CML stem cells from these first-round CML-affected mice and transplanted them into a second set of recipients. To our surprise, *Gdpd3*-deficient CML stem cells showed a significant decrease in their ability to induce CML, whereas WT-CML stem cells maintained this capacity in a second-round of BMT. These results demonstrate that Gdpd3 is critical for the long-term maintenance of CML stem cells in vivo.

Given that stem cell quiescence is vital for the maintenance of CML stem cells, we used in vivo BrdU incorporation assays to evaluate the cell cycle distribution of *Gdpd3*-deficient and WT-CML stem cells in CML-affected mice after a first-round of BMT. Importantly, the frequency of S-phase cells was strikingly increased among *Gdpd3*-deficient CML stem cells compared to WT-CML stem cells, indicating that *Gdpd3* loss activates the cell division of quiescent CML stem cells. Because sustained stem cell quiescence is the key to TKI resistance, we then investigated whether loss of Gdpd3 would affect TKI resistance in vivo. Strikingly, recipient mice that harbored *Gdpd3*-deficient CML stem cells and were treated with the TKI dasatinib showed a decrease in disease relapse even after a first-round of BMT. Thus, Gdpd3-mediated lysophospholipid metabolism in CML stem cells is critical for the maintenance of their quiescence and thus TKI resistance in vivo.

### 2.2. Lipidomics Analyses of the Gdpd3-Deficient CML Cells

Researchers have used numerous animal models and human patient samples to explore the roles of lysophospholipids in human diseases. For example, enforced transgenic expression of the *Atx* gene encoding the lysophospholipase D enzyme Autotaxin promoted tumor cell metastasis in a mouse model of breast cancer [22,23]. In ascites of human gastric cancer patients, lysophospholipids such as lysophosphatidyl serine (LPS) and lysophosphatidyl glycerol (LPG) were elevated [24]. Several LPAs were found to be increased in the plasma of acute coronary syndrome patients as well as in lumbar spinal cord stenoses in a rat model of cauda equina compression [25,26]. However, whether lysophospholipid metabolism is crucial in normal tissue stem cells and/or cancer stem cells has yet to be reported. Because Gdpd3 has lysophospholipase D activity that generates LPAs, we performed comparative lipidomics analyses of bone marrow CML stem cells from WT- and *Gdpd3*-deficient CML-affected mice. We observed that the mutant CML stem cells showed decreased LPAs compared to WT-CML stem cells [14]. These results suggest that lysophospholipid metabolism is indeed vital for CML stem cell functionality in vivo.

Lipid mediators and the signaling pathways they trigger play important roles in immune responses, inflammation and carcinogenesis [27,28,29]. Although phospholipids are known to be sources of several lipid mediators, including prostaglandins, leukotrienes and eicosanoids, it is not clear whether lysophospholipids and LPAs can also generate these lipid messengers. We conducted lipidomics analyses of 196 lipid mediators in total bone marrow CML cells and found that levels of prostaglandins, eicosanoids and a docosanoid were decreased in *Gdpd3*-deficient CML cells compared to WT-CML cells [14]. While it is still unknown exactly how Gdpd3 is involved in producing lipid mediators, these results suggest that at least some important messengers originate from lysophospholipid metabolism.

### 2.3. A Signaling Pathway That Regulates CML Stem Cell Quiescence

We have sought to define the underlying molecular mechanisms by which lysophospholipid metabolism affects stem cell quiescence and CML stemness. Because Gdpd3 loss activated the cell division of CML stem cells but attenuated their self-renewal capacity, we examined the phosphorylation of Akt and S6 ribosomal protein in WT- and *Gdpd3*-deficient LT-CML stem cells. Levels of phospho-Akt and phospho-S6 were increased in *Gdpd3*-deficient LT-CML stem cells compared to WT-LT-CML stem cells, indicating that *Gdpd3* loss activates the Akt–mTORC1 signaling pathway in CML stem cells. Consistent with this finding, Foxo3a was exported to cytoplasm and inactivated in *Gdpd3*-deficient LT-CML stem cells, in contrast to its nuclear (activated) localization in WT-LT-CML stem cells [14]. Thus, Gdpd3-mediated lysophospholipid metabolism regulates CML stem cell quiescence by suppressing the Akt–mTORC1 pathway and promoting nuclear Foxo3a localization. Collectively, our results indicate that lysophospholipid metabolism governs CML stemness in vivo in a manner that is independent of oncogenic BCR–ABL1 signaling.

### 2.4. Downstream Targets Underlying CML Stemness

**Figure 2:**
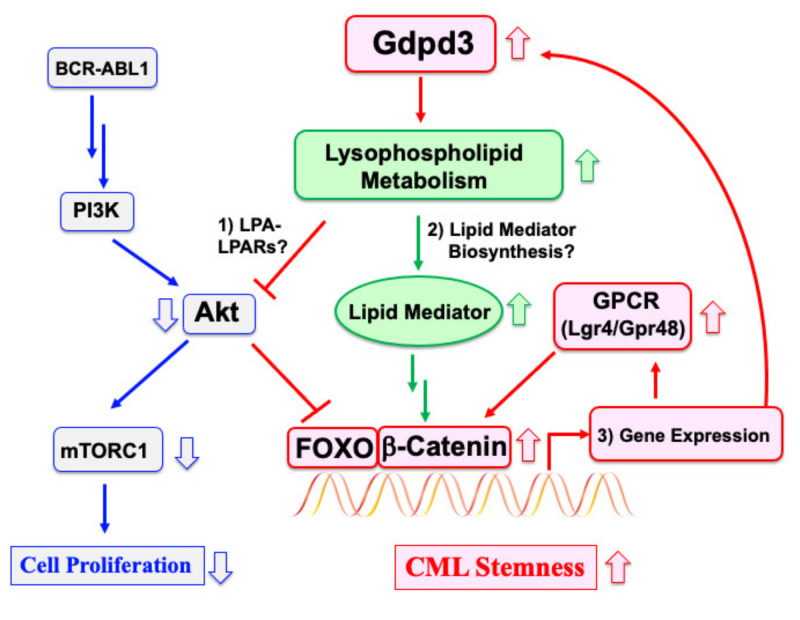
Regulation of CML stemness is independent of BCR–ABL1 oncogenic signaling. In the vast majority of mature CML cells, cell proliferation is driven by BCR–ABL1-mediated activation of the PI3K–Akt–mTORC1 signaling pathway. CML stem cells are able to maintain stem cell quiescence despite possessing the oncogene and so are TKI resistant. We have shown that Gdpd3 and lysophospholipid metabolism are essential for maintaining CML stem cell functions in vivo. Elevated lysophospholipid metabolism contributes to CML stemness by regulating an interaction between active Foxo3a and β-catenin (although the exact mechanism remains unclear). It is possible that Gdpd3-mediated lysophospholipid metabolism: (1) suppresses Akt via an LPA–LPARs pathway; (2) contributes to the biosynthesis of lipid mediators; and/or (3) participates in a gene expression program involving Gdpd3 and GPCRs by FOXO/β-catenin. Targeting any one of these elements of lysophospholipid metabolism specific to CML stem cells might provide fresh therapies to overcome disease relapse in many CML patients.

To understand gene expression patterns related to lysophospholipid metabolism in CML stem cells, we conducted comparative RNA-Seq analysis of WT- and *Gdpd3*-deficient LT-CML stem cells. We observed that expression levels of several genes encoding prototypical seven-transmembrane G-protein-coupled receptors (GPCRs) were attenuated in *Gdpd3*-deficient CML stem cells (Figure 2). Among these *GPCR* genes, mRNA levels of the *Lgr4/GPR48* gene, which encodes a leucine-rich repeat (LRR)-containing GPCR, were decreased in *Gdpd3*-deficient LT-CML stem cells compared to WT-LT-CML stem cells. Lgr4/GPR48 is known to be involved in the canonical Wnt/β-catenin signaling pathway [30,31,32], and β-catenin cooperates with active Foxo3a to regulate the metastasis of colon cancer cells [33]. β-catenin and Foxo3a are also known to be stemness factors for CML stem cells [12,34]. Thus, we investigated if active Foxo3a interacted with β-catenin in LT-CML stem cells. Such an interaction was readily detected in the nuclei of WT-LT-CML stem cells, but was dramatically reduced in LT-CML stem cells isolated from either our *Gdpd3*-deficient CML mouse model or an *Lgr4/Gpr48*-hypomorphic mutant CML mouse model [14]. We also determined if treatment in vitro with prostaglandin E2 (PGE2), which can activate β-catenin, could rescue the defective interaction between active Foxo3a and β-catenin in mutant LT-CML stem cells [35]. To our surprise, enforced treatment in vitro with PGE2 restored this interaction within the nuclei of *Lgr4/Gpr48*-hypomorphic mutant LT-CML stem cells, but not in *Gdpd3*-deficient LT-CML stem cells (where Foxo3a was inactivated in the cytoplasm). These results suggest that Gdpd3-mediated lysophospholipid metabolism may maintain the self-renewal capacity of CML stem cells by activating the stemness factors Foxo3a and β-catenin [14,15].

## 3. Additional Perspectives

### 3.1. Suppression of Akt by an LPA–LPARs Pathway

How exactly does Gdpd3-mediated lysophospholipid metabolism suppress the Akt–mTORC1 pathway and thus activate Foxo3a, leading to the quiescence essential for self-renewal capacity? (Figure 2). Taniguchi et al. performed a crystal structure analysis demonstrating that LPAs transduce their signaling by binding to the appropriate LPA receptors (LPARs) [36]. In our study, we found that expression levels of the *Lpar4/Gpr23* gene were decreased in *Gdpd3*-deficient LT-CML stem cells compared to WT-LT-CML stem cells. Igarashi et al. previously showed that bone marrow stromal cells isolated from *Lpar4*-deficient mice have a deficit in hematopoiesis-supporting capacity compared to those from WT mice [37]. It is therefore possible that LPA–LPAR4 binding may transduce signals between CML stem cells and stromal cells in their microenvironmental niche in a non-cell autonomous manner that preserves CML stemness. Further investigations are required to understand the precise mechanisms, but it is clear they must be independent of BCR–ABL1 signaling.

### 3.2. Functional Links between Lysophospholipid Metabolism and Lipid Mediator Biosynthesis

Although it has yet to be clarified how lysophospholipid metabolism is involved in the biosynthesis of downstream lipid mediators, several previous reports have established that certain lipid mediators and their downstream signaling targets are required for CML stemness. For example: (1) Murine CML stem cells isolated from mice lacking either *arachidonate 5-oxygenase (Alox5)* or *Alox15* display decreased self-renewal capacity in vivo [38,39]. (2) The administration of the anti-diabetic drug pioglitazone induces DMR in TKI-insensitive CML patients by activating the proliferator-activated receptor γ (PPARγ) signaling pathway and thereby eradicating CML stem cells [40]. (3) Treatment of CML-affected mice with PGE1 plus the TKI imatinib increases therapeutic benefit over treatment with imatinib alone. Indeed, treatment of recipient mice transplanted with CML stem cells with misoprostol, an agonist of the E-type proteinoid receptor-4 (EP4), has therapeutic effects [35]. (4) *Lpar3*-deficient female mice show decreased *Cox2* mRNA and defects in embryo implantation, a phenotype similar to that of *Cox2*-deficient mice lacking the Cox2 enzyme essential for generating prostaglandins [41,42]. Treatment of *Lpar3*-deficient female mice with PGE2 or cPGI (a stable PGI2 analogue) partially rescued these defects [41]. (5) In our study, *Gdpd3*-deficient CML stem cells showed decreased PGE2 levels, and in vitro treatment of *Lgr4*-hypomorphic mutant CML stem cells with PGE2 restored the interaction between active Foxo3a and β-catenin [14]. Collectively, these reports reinforce our contention that stimulation of lipid mediators underlying lysophospholipid metabolism promotes CML stemness.

Our knowledge is still limited as to precisely how lysophospholipids are involved in the production of the lipid mediators needed to maintain CML stem cells. It is possible that lysophospholipids are direct sources of such lipid mediators, and/or that lysophospholipids regulate the expression of genes critical for lipid mediator biosynthesis. To distinguish between these possibilities, we should determine the substrates and products of the metabolic reaction governed by Gdpd3 by using ^13^C-stable isotopic metabolite tracing experiments. The results of these studies will shed much-needed additional light on how lysophospholipid metabolism is linked to vital lipid mediators in CML stem cells.

### 3.3. A Gene Expression Program Involving Gdpd3 and GPCRs by FOXO/β-Catenin

Another unanswered question in the field is how lysophospholipid metabolism regulates the transcription of *GPCR* mRNAs. We observed that *Gdpd3*-deficient LT-CML stem cells showed decreased expression of *GPCR* genes, including *Lgr4/GPR48*, compared to WT-LT-CML stem cells [14]. Interestingly, Lgr4/GPR48 is involved in canonical Wnt/β-catenin signaling as a receptor for R-Spondins [30,31,32]. Recently, Salik et al. reported that R-Spondin-3 and Lgr4/GPR48 regulate self-renewal capacity in acute myelogenous leukemia (AML) stem cells in vivo [43], paralleling our finding that Lgr4/GPR48 is essential for the maintenance of CML stem cells in mice [14]. We also showed that LT-CML stem cells from *Lgr4/GPR48*-hypomorphic mutant mice had a defect in active Foxo3a/β-catenin interaction that could be rescued in vitro by PGE2. However, this treatment could not restore Foxo3a/β-catenin interaction lost due to lack of Gdpd3. Notably, Beulac et al. reported that Foxo3 induces *Gdpd3* expression in a mouse model of noise-induced hearing loss [44]. These data suggest the existence of a gene expression program involving Gdpd3 and GPCRs by Foxo3a/β-catenin (Figure 2). Future work should clarify the transcriptional targets of the putative Foxo3a/β-catenin complex that governs gene expression patterns required for CML stem cell functions in vivo.

## 4. Conclusions

In this review, I have attempted to put into a broader biological context our recent findings on the role of lysophospholipid metabolism in general, and Gdpd3 in particular, in CML stem cells in vivo. Importantly, healthy Gdpd3-deficient mice show no obvious defects [14,45], suggesting that specific inhibition of Gdpd3-mediated lysophospholipid metabolism may be a viable means of therapeutically targeting CML stem cells without generating harmful side effects in normal tissue stem cells. Future work should determine if lysophospholipid metabolism is critical in other types of hematological cancer stem cells and/or in solid tumors. Regulating pathways within the lysophospholipid metabolome may open up new avenues for maintaining the quiescence of cancer stem cells, thereby potentially preventing disease relapse in cancer patients.
